# Role of patient and tumor characteristics in sentinel lymph node metastasis in patients with luminal early breast cancer: an observational study

**DOI:** 10.1186/s40064-016-1720-9

**Published:** 2016-02-03

**Authors:** Nicla La Verde, Elena Biagioli, Chiara Gerardi, Andrea Cordovana, Chiara Casiraghi, Irene Floriani, Elena Bernardin, Gabriella Farina, Serena Di Cosimo, Maria Chiara Dazzani, Giorgio Gherardi

**Affiliations:** Oncology Department, Fatebenefratelli and Oftalmico Hospital, Corso di Porta Nuova 23, 20121 Milan, Italy; IRCCS - Istituto di Ricerche Farmacologiche Mario Negri, Via La Masa 19, 20156 Milan, Italy; IRCCS - Istituto Nazionale Tumori, Via Venezian 1, 20133 Milan, Italy

## Abstract

Predicting the risk of sentinel lymph node (SLN) metastasis is important for clinical decision-making in the setting of early breast cancer (EBC). This study is aimed to identify tumor and patient characteristics that influenced the SLN metastatic involvement, with a focus on luminal subtypes. An observational study including women treated for EBC from 2005 to 2013 was conducted. Regression analyses were used to assess the association between SLN metastasis and age, menopausal status, tumor size, histological grading, presence of extensive “in situ” carcinoma components, lymphovascular invasion (LVI), and expression of Ki-67, hormone receptors, and HER2. Of 345 women, 84 (24.3 %) had at least one SLN metastasis; 63.1 % were macrometastases. Among all patients, 31.6 % exhibited LVI. In univariate analyses, tumor size, histological grade, and LVI were associated with SLN metastasis. The multivariate model confirmed only the association between LVI and SLN status (OR 3.27, 95 % CI 1.85–5.68; p < 0.0001). Luminal subtypes were detected in 86.1 % of women. In this subgroup, the multivariate model confirmed a significant relationship between LVI and SLN status (OR 3.47, 95 % CI 1.90–6.33; p < 0.0001). Since a proper histopathological assessment of LVI is not possible prior to surgery, this factor cannot be used to guide decisions on performing SLN biopsies. Nevertheless, when a SLN biopsy is refused or contraindicated, an LVI assessment on an excisional biopsy of the tumor could facilitate prognosis determination and treatment management.

## Background

Early-stage breast cancer (EBC) is confined to the breast with or without regional lymph node involvement (NHSBSP and RCPath 2005). In developed countries, EBC is treated with surgery, local irradiation, and adjuvant systemic therapy, when needed. This treatment provides long term survival in over 80 % of women diagnosed with EBC (Coleman et al. 2008).

In the treatment of EBC, sentinel lymph node dissection (SLND) has overcome the need for axillary lymph node dissection (ALND) in patients whose sentinel node (SLN) is free of metastasis (Veronesi et al. 2003). In fact, although ALND is thought to be the most accurate method for assessing disease spread to the lymph nodes, the anatomic disruption caused by ALND causes significant complications and side effects which can compromise functionality and quality of life (Lyman et al. 2005). Recently, accumulating evidence has shown that ALND could also be avoided in patients with EBC that involved SLNs ranging from micro- to macro-metastatic disease (Galimberti et al. 2013) in up to two lymph nodes (Giuliano et al. 2011). In addition, multivariate analyses have clearly demonstrated that only tumor grade (measured by the modified Bloom–Richardson histological score) and age were significantly associated with locoregional recurrence (Giuliano et al. 2010) and that tumor size and tumor grade could significantly predict disease-free survival (DFS), irrespective of the use of ALND (Giuliano et al. 2011). According to those results, in the near future, prognosis may be predicted more accurately with alternative, intrinsic biological tumor features, obtained with conventional histopathological, immunohistochemical, or molecular biological characterizations, which can assess the potential of local and systemic tumor aggressiveness. These approaches may be more informative than the conventional T (tumor size), N (lymph node involvement) and M (metastasis) classifications for decisions concerning the optimal choice of locoregional and systemic treatments. Thus, it has become increasingly desirable to devise a means to identify patients with EBC that can be treated safely without invasive, mutilating axillary procedures, based on patient clinical parameters and biological features intrinsic to the tumor.

An important goal for this category of patients is to define the actual risk of axillary node metastasis, based on tumor features. In fact, several studies have attempted to achieve this goal to date, but they have produced somewhat different, and sometimes conflicting, results (Gill et al. 2006; Gonzalez-Vela et al. 1999; Rivadeneira et al. 2000; Viale et al. 2005; Yoshihara et al. 2013). In the present study, we reviewed a case series of patients with EBC with the aim of identifying key primary tumor characteristics and patient clinical features that might influence SLN metastasis, with a special focus on luminal subtypes of breast cancer. We discussed our results from the perspective of their potential clinical implications, in light of the most recent literature.

## Methods

This observational study included women treated for EBC at Azienda Ospedaliera Fatebenefratelli and Oftalmico in Milan, Italy. The study protocol adhered to the tenets of the Declaration of Helsinki and was approved by the Institutional Review Board. Data was handled according to current Italian legislation on observational studies.

The primary aim of this study was to evaluate associations between patient and tumor characteristics at diagnosis and the development of metastases in the SLN. Secondary aims were to evaluate the aforementioned associations in the subgroup of patients with the luminal subtypes of EBC, and to evaluate the influence of patient and tumor characteristics on disease free survival (DFS) and overall survival (OS).

### Patients

Women with EBC that underwent breast surgery (mastectomy, lumpectomy, or quadrantectomy) and a SLN biopsy (SLNB) were considered eligible.

Inclusion criteria were: histologically confirmed, invasive breast cancer; tumor size, less than 5 cm; and lack of clinical/ultrasound evidence of axillary node metastatic involvement. Exclusion criteria were: male gender; previous breast cancer (either invasive or “in situ”); inflammatory breast cancer presentation; previous neoadjuvant chemotherapy or hormone therapy; synchronous metastasis at diagnosis; bilateral breast cancer; or multicentricity of the tumor.

### Pathology

Pathological examinations of SLN were performed postoperatively on permanent paraffin sections in all cases. Immediately after excision, fresh SLNs were sent to the laboratory, then immediately fixed, unsliced, in 4 % buffered formaldehyde. After 2–4 h of fixation, nodes were cut into multiple slices, 1-mm thick, fixed overnight, and embedded in paraffin, according to routine protocols. For microscopic evaluation, paraffin sections were collected at about 200-micron intervals until complete examination of the lymph node slices. At each level, one additionally collected paraffin section was preliminarily left unstained to provide a section for immunostaining, when required, based on a routine microscopic scrutiny of the corresponding hematoxylin and eosin stained slide.

All breast tumors were extensively sampled according to the National Health Services Royal College of Pathologists Recommendations Protocol (NHSBSP and RCPath 2005). Tumor size was assessed based on the largest diameter (mm) in the invasive component. Histologic tumor types were categorized as ductal, lobular, and mixed. The mixed category included pure tubular, pure colloid (mucinous), typical medullary carcinomas, etc. Microscopic grading was assigned according to the Nottingham modification of the Bloom–Richardson system (Ellis et al. 2006). Lobular carcinomas were assigned a nuclear grade according to Fisher’s system (Fisher et al. 1986). Lymphovascular invasion (LVI) was recorded when at least one neoplastic thrombus was detected in a peritumoral lymphatic vessel, but its extent was not graded. The ductal or lobular component of a carcinoma “in situ” was classified as “extensive” when it represented >25 % of the tumor. Estrogen receptor (ER) and progesterone receptor (PgR) status were evaluated with standard immunohistochemical techniques, and at least 1 % nuclear staining was required to deem the specimen positive. The proliferative cellular compartment of the tumor was measured semi-quantitatively, based on immunostaining for Ki-67. Primary antibodies for specific detection of ER, PgR, and Ki-67 were derived from clones SP1, 1E2, and 30-9, respectively (Ventana Medical Systems Inc. Tucson, AZ, USA). HER2 testing was performed immunohistochemically, with the primary antibody derived from clone CB11 (Cell Marque Corp., Rocklin, Ca, USA) and, when samples received scores of 2+, we used fluorescence in situ hybridization, according to the American Society of Clinical Oncology/College of American Pathologists Guidelines (Wolff et al. 2007). Tumor molecular subtypes were classified according to Maisonneuve et al. In particular, luminal A–like tumors were ER-positive and HER2-negative, with low (<14 %) or intermediate (14–19 %) Ki-67 expression, and high PgR levels (≥20 %). Luminal B–like (HER2-negative) tumors were ER-positive and HER2-negative, with intermediate Ki-67 expression (14–19 %) and low PgR levels (<20 %), or with high Ki-67 expression (≥20 %) (Maisonneuve et al. 2014). However, Maisonneuve et al. did not provide modifications for defining the “luminal B (HER2-positive)”, “HER2-positive”, or “triple-negative” breast cancer subtypes; therefore, we classified these cases according to the St. Gallen Consensus (Goldhirsch et al. 2013).

### Statistical analysis

Patient and tumor characteristics are expressed as absolute and relative frequencies for categorical variables. They are expressed as mean, standard deviation (SD), minimum and maximum values for continuous variables.

Logistic regression models were performed to evaluate whether SLN status was influenced by age, menopausal status, number of sentinel nodes excised, tumor size, molecular subtype (including quantitative evaluation of Ki-67, ER, PgR, and HER2 expression), histological grading, LVI, and the presence of an extensive “in situ” carcinoma component. First, we used univariate models to identify independent variables, then, we used a multivariate model that included variables from the univariate analyses that were related (p < 0.10) to the SLN status. The results are expressed as odds ratios (ORs) with their respective 95 % confidence intervals (95 % CIs).

The DFS and OS were described with the Kaplan–Meier method.

The influence of patient and tumor characteristics on DFS was analyzed with Cox proportional-hazards regression models. First, we performed univariate analyses, then we constructed a multivariate model with the same approach of primary endpoint. Results were expressed as hazard ratios (HRs) and relative 95 % CIs. Analyses were performed with SAS statistical software (version 9.4).

## Results

### Patient and tumor characteristics

We evaluated 505 consecutive patients for study inclusion. All patients had undergone breast surgery and SLNB between January 1st, 2005 and September 30th 2013. Of these, 160 were excluded for the following reasons: in situ carcinoma (67 cases); previous breast cancer (19 cases); synchronous metastasis (6 cases); bilateral breast cancer (5 cases); previous neoadjuvant chemotherapy (4 cases); papillomatosis (3 cases); male (1 case) and loss to follow-up after surgery (55 cases).

We included 345 eligible women with a mean age of 61 years (SD 11.3, range 29.9–87.7). Of these, 78.8 % were postmenopausal and 84.6 % had undergone quadrantectomy. Most women (n = 227, 65.8 %) had only one SLN removed, but the overall mean number of SLNs removed was 1.5 (range 1–7). A total of 84 patients (24.3 %) had at least one positive SLN, including 63.1 % macrometastases and 36.9 % micrometastases (Table 1).

**Table 1:** Patient characteristics

Patients—N (%)	345 (100.0)
Age at surgery, years	
Mean (SD)	61.0 (11.3)
Min–max	29.9–87.7
Menopausal status—N (%)	
Pre	73 (21.2)
Post	272 (78.8)
Type of surgery—N (%)	
Mastectomy	44 (12.8)
Quadrantectomy	292 (84.6)
Nodulectomy	9 (2.6)
Number of excised sentinel nodes per patient	
Mean (SD)	1.5 (0.9)
Min–max	1–7
Number of sentinel nodes excised, distribution—N (%)	
1	227 (65.8)
2	77 (22.3)
3	28 (8.1)
4	8 (2.3)
≥5	5 (1.4)
Number of positive sentinel nodes per patient	
Mean (SD)	1.2 (0.5)
Min–max	1–4
Sentinel node status	
Patients with negative sentinel nodes—N (%)	*261 (75.7)*
Isolated tumoral cells	33 (12.6)
No Isolated tumoral cells	228 (87.4)
Patients with positive sentinel nodes—N (%)	*84 (24.3)*
Micrometastasis	31 (36.9)
Macrometastasis	53 (63.1)
Axillary dissection—N (%)^a^	65 (18.8)
Number of resected nodes per patient	
Mean (SD)	15.4 (5.5)
Min–max	1–31
Number of positive nodes per patient	
Mean (SD)	1.8 (3.5)
Min–max	0–18

*Min–max* minimum and maximum values

^a^65 patients underwent axillary dissection

Most patients (n = 261, 75.7 %) had small tumors (<20 mm); the mean tumor size was 15.7 mm (range 1.0–50.0 mm). Histology showed that 57.4 % of tumors had low Ki-67 expression (range 0–13) and 70.7 % had a low histological grade (Grade 1 or Grade 2). LVI was detected in 31.6 % of all patients. The luminal subtype was detected in 297 women (86.1 %) (Table 2).

**Table 2:** Tumor characteristics

Histology—N (%)	
Ductal	199 (57.7)
Lobular	56 (16.2)
Mixed	56 (16.2)
Other^a^	34 (9.9)
Average tumor size (mm)	
Mean (SD)	15.66 (7.90)
Min–max	1.00–50.00
Tumor size, distribution—N (%)	
T < 20 mm	261 (75.7)
20 mm < T ≤ 50 mm	84 (24.3)
Site of tumor—N (%)	
QII	21 (6.1)
QSI	46 (13.3)
QSE	225 (65.2)
QIE	45 (13.0)
CENTRAL	8 (2.3)
Ki-67—(% expression)	
Mean (SD)	15.02 (10.18)
Min–max	5.00–80.00
Distribution of Ki-67 expression levels—N (%)	
Low (0–13 % of cells)	198 (57.4)
Intermediate (14–9 % of cells)	60 (17.4)
High (≥20 % of cells)	87 (25.2)
Patients with ER positivity—N (%)	297 (86.1)
ER positivity per patient—(% expression)	
Mean (DS)	65.23 (33.35)
Min–max	0.00–100.00
Patients with PGR positivity—N (%)	275 (79.7)
PgR ≥20 % expression	238 (69.0)
PgR positivity per patient—(% expression)	
Mean (SD)	50.83 (36.59)
Min–max	0.00–100.00
Patients with HER2 positivity—N (%)	45 (13.0)
Distribution of histological grades—N (%)	
Grade 1	69 (20.0)
Grade 2	175 (50.7)
Grade 3	101 (29.3)
Tumor subtypes—N (%)	
Patients with luminal subtype	*297 (86.1)*
luminal A	225 (75.8)
luminal B	72 (24.2)
Patients with HER 2 subtype	*21 (6.1)*
Patients with triple negative subtype	*27 (7.8)*
Patients with LVI—N (%)	109 (31.6)
Patients with extensive DCIS/LCIS	98 (28.4)

*Min–max* minimum and maximum values, *LVI* LymphoVascular Invasion, *DCIS* Ductal Carcinoma In Situ, *LCIS* Lobular Carcinoma In Situ

^a^Other histological descriptions: mucinous; tubular; apocrine; medullary; papillary

After surgery, 83.5 % of patients received endocrine therapy, 30.4 % underwent chemotherapy, 9.3 % received immunotherapy with trastuzumab, and 76.2 % underwent radiotherapy.

### Association between patient/tumor characteristics and SLN status in the overall population

Univariate analyses showed that tumor size, histological grade, and LVI were associated with the presence of SLN metastasis. The multivariate model confirmed that only LVI had a significantly negative prognostic association with SLN status. Compared to those without LVI, women with LVI had a three-fold higher risk of SLN metastasis (OR 3.27, 95 % CI 1.85–5.68; p < 0.0001). Table 3 shows the results from logistic models.

**Table 3:** Factors associated with sentinel node status

	Overall sample (N = 345)				Luminal subgroup (N = 297)			
	Univariate OR (95 % CI)	p value	Multivariate OR (95 % CI)	p value	Univariate OR (95 % CI)	p value	Multivariate OR (95 % CI)	p value
Age (increase of 10 years)	0.98 (0.79–1.22)	0.880			0.93 (0.74–1.18)	0.552		
Menopausal status (pre vs. post)	1.23 (0.68–2.20)	0.495			1.28 (0.68–2.42)	0.450		
Number of excised sentinel nodes (continuous variable)	1.06 (0.81–1.39)	0.656			1.08 (0.80–1.44)	0.627		
Histology								
Ductal (reference)	1	0.127			1	0.205		
Lobular	1.02 (0.51–2.03)				1.06 (0.52–2.14)			
Mixed	1.45 (0.76–2.77)				1.24 (0.63–2.46)			
Others	0.30 (0.09–1.01)				0.13 (0.02–0.98)			
Tumor size (increase of 10 mm)	1.56 (1.14–2.13)	*0.005*	1.20 (0.86–1.68)	0.289	1.71 (1.19–2.45)	*0.004*	1.22 (0.84–1.78)	0.307
Subtype								
Luminal A (reference)	1				1	0.215		
Luminal B	1.39 (0.81–2.37)	0.232			1.41 (0.82–2.40)			
HER2	2.16 (0.84–5.57)	0.111						
Triple negative	0.46 (0.13–1.60)	0.222						
Histological grade								
Grade 1 (reference)	1		1		1		1	
Grade 2	2.88 (1.28–6.47)	*0.010*	2.28 (0.99–5.24)	0.054	2.67 (1.18–6.01)	*0.018*	2.08 (0.89–4.83)	0.089
Grade 3	2.92 (1.24–6.88)	*0.014*	1.81 (0.73–4.48)	0.198	3.08 (1.23–7.68)	*0.016*	2.15 (0.82–5.61)	0.119
Presence of LVI (yes vs. no)	3.81 (2.27–6.37)	*<.0001*	3.27 (1.89–5.68)	*<.0001*	4.21 (2.40–7.36)	*<.0001*	3.47 (1.90–6.33)	*<.0001*
Presence of extensive DCIS/LCIS (yes vs. no)	1.09 (0.64–1.87)	0.751			1.28 (0.72–2.26)	0.396		

*LVI* LymphoVascular Invasion, *DCIS* Ductal Carcinoma In Situ, *LCIS* Lobular Carcinoma In Situ

### Association between patient/tumor characteristics and SLN status in the luminal (A and B) subgroup

This analysis included 297 women diagnosed with luminal (A or B) breast cancer. Among all luminal cases, a univariate analysis showed that a positive SLN was significantly associated with tumor size (OR for a 10 mm increase: 1.71, 95 % CI 1.19–2.45; p = 0.004), histological grade (Grade 2 vs. Grade 1: OR 2.67; 95 % CI 1.18–6.01; p = 0.018; Grade 3 vs. Grade 1: OR 3.08, 95 % CI 1.23–7.68; p = 0.016), and LVI (OR 4.21; 95 % CI 2.40–7.36; p < 0.0001). The multivariate model showed that only the presence of LVI significantly affected SLN status (OR 3.47, 95 % CI 1.90–6.33; p < 0.0001; Table 3).

### Survival analysis

**Fig. 1:**
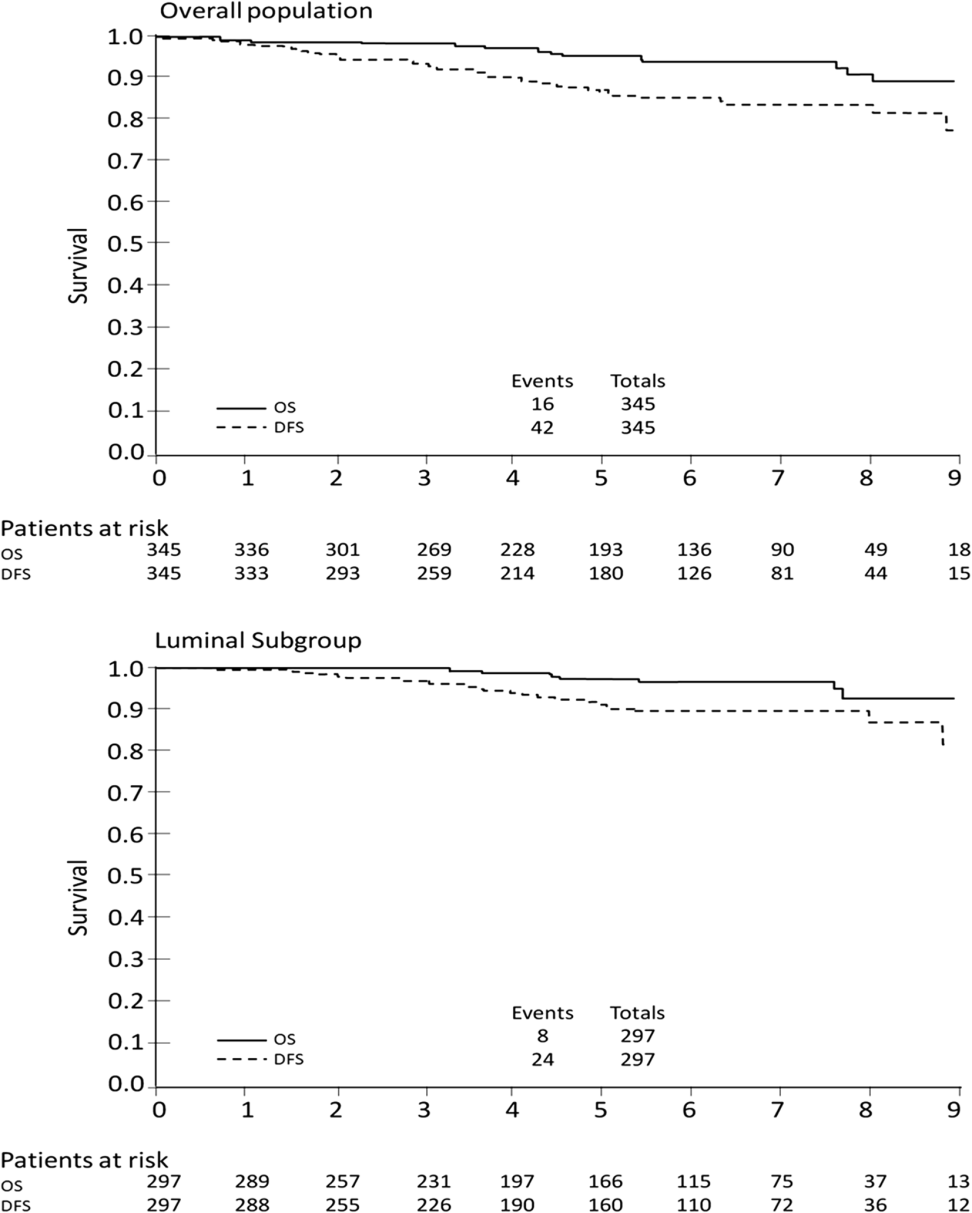
Kaplan Meier curves of DFS and OS

We analyzed survival in the overall sample and in the luminal subgroup (Fig. 1).

The median follow-up of the overall population (n = 345) was 5.4 years (interquartile range 3.3–7.2 years). At the time of analysis, 38 (11.0 %) women relapsed and 16 (4.6 %) died; a total of 42 (12.2 %) patients either relapsed or died. For patients with luminal breast cancer (n = 297), these figures were 21 (7.1 %), 8 (2.7 %), and 24 (8 %), respectively.

A univariate analysis of the overall sample showed that DFS was influenced by age, tumor size, histological grade (Grade 3 vs. Grade 1), molecular subtype (HER 2 vs. luminal A; triple negative vs. luminal A), and LVI. In the multivariate Cox proportional-hazards regression model, the only variable confirmed to play a role in patient prognosis was the triple negative subtype. Patients with triple negative tumors had a six-fold higher risk of relapse or death than patients with luminal A tumors (HR: 5.94, 95 % CI 2.09–16.85; p = 0.0008; Table 4). A univariate analysis in the luminal subgroup also showed that patient prognosis was associated with a SLN positive for metastasis and the variables mentioned above, which were identified for the overall sample. A multivariate model was not performed, due to the low number of observed events (24 relapses or deaths).

**Table 4:** Factors associated with Disease Free Survival—overall population

	Univariate HR (95 % CI)	p value	Multivariate HR (95 % CI)	p value
Age (increase of 10 years)	0.74 (0.56–0.97)	*0.027*	0.83 (0.63–1.10)	0.194
Tumor size (increase of 10 mm)	1.33 (0.98–1.81)	*0.064*	1.01 (0.68–1.49)	0.961
Histological grade				
Grade 1 (reference)	1		1	
Grade 2	2.03 (0.59–7.01)	0.264	1.84 (0.52–6.56)	0.347
Grade 3	5.73 (1.73–19.04)	*0.004*	2.39 (0.62–9.17)	0.205
Subtype				
Luminal A	1			
Luminal B	2.11 (0.95–4.73)	0.067	1.62 (0.69–3.79)	0.267
HER2	5.68 (2.18–14.74)	*0.0004*	3.88 (1.34–11.25)	0.013
Triple negative	9.36 (4.04–21.67)	*<.0001*	5.94 (2.09–16.85)	*0.0008*
SLN positivity				
No metastasis	1			
Positive metastasis	1.68 (0.89–3.16)	0.108		
SLN pattern				
No metastasis	1			
Micrometastasis	1.72 (0.71–4.18)	0.230		
Macrometastasis	1.65 (0.77–3.51)	0.193		
Presence of LVI (yes vs. no)	1.84 (0.98–3.44)	*0.057*	1.19 (0.60–2.33)	0.622

*SLN* Sentinel Lymph Node, *LVI* LymphoVascular Invasion

## Discussion

Predicting the risk of SLN metastatic involvement is an important aspect of clinical decision-making in the setting of EBC.

A previous systematic review of the literature, which included 290 papers, was focused on prognostic factors of axillary lymph node involvement. They failed to find any association between nodal status and tumor size, grading, multifocality, LVI, neoangiogenesis, hormone receptor status, or selected protein and genetic markers. However, those authors emphasized the limitations of their findings: many of the included studies were retrospective, had small sample sizes, and did not implement a fully adequate statistical approach (Patani et al. 2007). The present study was conducted with a large series of patients with EBC. We found that LVI was an independent risk factor for SLN metastatic involvement. The association between peritumoral LVI and the incidence of SLN metastatic involvement has been analyzed in previous studies. Viale et al. (Viale et al. 2005) reported that the presence of LVI combined with a large tumor size, ductal histotype, presence of multifocality, and high PgR expression could predict SLN metastatic involvement. Aitken et al. (Aitken and Osman 2010) found that lymph node metastases was most strongly predicted by a tumor size >50 mm (OR 2.33), followed by the presence of LVI (OR 1.33). In contrast, our data showed that only peritumoral LVI was associated with SLN metastatic involvement. This discrepancy may be partially explained by differences in the populations examined, particularly in the above-mentioned papers, which included larger tumors and more advanced disease stages than those included in the present study.

In another study, Yoshihara et al. evaluated patients and tumor factors associated with axillary lymph node metastasis on cT1-T2 invasive breast cancer without a specific analysis on SLN metastasis. In their cohort of 1300 patients, nodal involvement was associated with the presence of LVI (p < 0.0001), large tumor size (p < 0.0001), ALND (p = 0.0003), retroareolar and lateral tumor locations in the breast (p = 0.0019), and the presence of multiple foci (p = 0.0155) (Yoshihara et al. 2013). More recently, the problem of staging the axilla has been discussed from another point of view; i.e. predicting the risk of locoregional recurrence, based on tumor and patient characteristics. Galimberti et al. stated that tumor size and tumor grade were predictors of DFS, but axillary dissection versus no axillary dissection was not a significant factor (Galimberti et al. 2013). Giuliano et al., demonstrated in their study that ALND could be avoided in selected patients with positive SLN. They identified factors (other than SLN status) that could predict locoregional recurrences, including the modified Bloom–Richardson histological grading score and age (Giuliano et al. 2010). Our data also provided further evidence that LVI could predict a high risk of SLN metastasis in luminal type EBC. These findings support previous studies that showed that LVI, combined with tumor size and tumor grade, influenced axillary lymph node involvement (Bevilacqua et al. 2007; Klar et al. 2009); LVI has also been shown to predict a high risk of concomitant metastasis in non-sentinel lymph nodes (Kwon et al. 2011; Liu et al. 2014). Moreover, the presence of peritumoral LVI was shown to play a role in the prognosis of patients with breast cancer, irrespective of SLN status. Furthermore, in patients with T1 tumors, the risk of death due to breast carcinoma or tumor recurrence was roughly two- to three-fold higher in women with lymphatic emboli compared to women without lymphatic peritumoral emboli (Bettelheim et al. 1984; Rosen et al. 1981; Roses et al. 1982).

When we considered the subtype of luminal breast cancers, we found that only the detection of peritumoral LVI could predict the probability of SLN involvement; neither histological grade nor tumor size seemed to impact the risk of metastatic involvement. However, detecting peritumoral LVI requires a histopathological examination of the tumor in its entirety (i.e., on an excised biopsy sample). Unfortunately, a core biopsy sample is not the optimal procedure for identifying or excluding with certainty the presence of peritumoral LVI, because peritumoral areas are not sampled, and only a small part of the tumor is examined. Consequently, our data are provocative because, in the specific setting of luminal types of EBC, the conventional preoperative diagnostic work-up may not provide a reliable assessment of LVI, thus, it lacks any role in predicting the risk of SLN metastasis This latter statement would confirm the conclusions of Jones et al., who studied the prognostic role of breast cancer subtypes in nodal involvement; they found significant associations between different breast cancer subtypes and age, tumor stage, histology, method of detection, and race, but no associations with nodal involvement. Those authors concluded that the breast cancer subtype may not be a useful prognostic factor for decisions concerning the local regional management of EBC (Jones et al. 2013). In any case, this controversial puzzling issue is soon likely to become obsolete, due to data provided by the ongoing SOUND study. That study is being conducted to evaluate outcome in patients that received no axillary procedure, except an ultrasound examination, with or without fine needle aspiration (Gentilini and Veronesi 2012). The goal of that study, which incidentally, may be supported by our present observations, is to evaluate patient outcome in the absence of a SLN examination; thus, they are challenging the paradigm of examining the SLN in all patients with EBC.

Finally, our survival analyses showed that patients with triple negative tumors had close to six-fold greater risk of relapse or death than patients with luminal A tumors. This observation confirmed the well known data that indicate the magnitude of the risk of a poor prognosis for these patients (Foulkes et al. 2010). However, unlike the overall population, in the luminal subgroup, SLN metastasis appeared to influence DFS; the presence of SLN metastasis doubled the risk of relapse or death compared to luminal EBC without SLN metastasis.

In conclusion, currently, axillary lymph node metastasis is the most important prognostic factor in EBC. The results presented herein may be useful for managing select groups of patients who, due to comorbidities or refusal, have avoided surgical interventions in the axilla. Knowledge of axillary status may influence oncologists in selecting and prescribing adjuvant systemic therapy (Montemurro et al. 2012).Thus, the information provided by our study may be used to inform treatment decisions; e.g., in discussing with patients the benefits of adjuvant chemotherapy in cases of luminal breast cancer with LVI, irrespective of axillary status.

Moreover, our findings have suggested that no ultimate decision on the management of the axilla should be based only on a diagnostic core-biopsy of the primary tumor, due to the limited ability to assess LVI. This study further confirmed the importance of biological information in the research for low impact procedures. The results of this study underline the need to improve the accuracy of existing diagnostic tests and the need to explore new strategies with high accuracy, based on the mechanisms involved in breast cancer development.
